# Membranous Fetal Vessels Mimicking Amniotic Band Syndrome: Furcate Cord Insertion in a Bilobed Placenta

**DOI:** 10.1055/a-2973-7161

**Published:** 2026-10-30

**Authors:** Abbey Pfister Donahue, Sophia Brancazio, Kristen Quinn, Talla Widelock, Matthew Zuber

**Affiliations:** 1 Department of Maternal Fetal Medicine 8674 Novant Health, Inc.Winston-SalemNorth CarolinaUnited States; 2 Section of Maternal-Fetal MedicineDepartment of Obstetrics and Gynecology 22442 Atrium Health Carolinas Medical CenterCharlotteNorth CarolinaUnited States; 3 Section of Maternal-Fetal MedicineDepartment of Obstetrics and Gynecology 12279 Wake Forest University School of MedicineWinston-SalemNorth CarolinaUnited States

**Keywords:** furcate cord insertion, umbilical vessels, color doppler, amniotic band, prenatal diagnosis

## Abstract

**Objective** Membranous fetal vessels may mimic amniotic band syndrome, creating diagnostic uncertainty. We describe a case in which color Doppler redirected the diagnosis from a presumed amniotic band toward furcate cord insertion in a bilobed placenta.

**Study Design** Single case report of a 33-year-old gravida 3 para 2 evaluated with serial transabdominal and transvaginal ultrasound with color Doppler, correlated with gross placental examination after delivery.

**Results** Ultrasound at 19 ^6/7^ weeks demonstrated a membranous sheet spanning the lower uterine segment without direct fetal involvement, initially suggesting amniotic band syndrome. Color Doppler revealed fetal vessels within the membrane, raising concern for abnormal cord insertion and vasa previa. Transvaginal color Doppler localized these vessels more than 3 cm from the internal cervical os, arguing against type I vasa previa. Surveillance and antenatal testing remained reassuring, and a cesarean at 37 ^3/7^ weeks yielded a healthy neonate. Gross examination confirmed a bilobed placenta with furcate separation of umbilical vessels inserting velamentously into each lobe and converging into a three-vessel cord.

**Conclusion** Membranous fetal vessels from furcate cord insertion may mimic both amniotic band syndrome and vasa previa. Color Doppler interrogation of any intrauterine band-like structure is essential to establish the correct diagnosis and guide management.

**Key Points**

- Furcate cord insertion can mimic amniotic bands.
- Color Doppler excludes a true amniotic band.
- Vessel flow in a band suggests cord insertion.
- Exposed vessels risk compression or rupture.

## Introduction

Furcate umbilical cord insertion is a rare placental abnormality in which the umbilical vessels separate and lose their protective Wharton’s jelly before reaching the placental surface, coursing unsupported along the fetal membranes before insertion. Pathologic series estimate a prevalence of approximately 0.1 to 0.16% of pregnancies, but prenatal diagnosis is uncommon, and the condition is likely underrecognized. 1 2 Because the exposed vessels lack the mechanical protection of Wharton’s jelly, they may be vulnerable to compression or rupture, and intrauterine fetal demise attributable to vessel rupture has been reported. 1

On prenatal ultrasound, membranous fetal vessels associated with abnormal cord insertion can produce a thin, linear, echogenic intrauterine structure that closely resembles an amniotic band on grayscale imaging. Linear intrauterine structures are frequently presumed to represent amniotic bands; however, true amniotic band involvement of the umbilical cord, while rare, is potentially catastrophic and often diagnosed only after fetal demise. Failure to distinguish a vascularized membrane from a true amniotic band may lead to either inappropriate reassurance or unnecessary alarm, and the distinction carries substantially different management implications. 3

We report a case in which a second-trimester membranous intrauterine structure initially raised concern for amniotic band syndrome but was shown on color Doppler to contain fetal vessels, ultimately confirmed at delivery to represent furcate cord insertion in a bilobed placenta. This case underscores the essential role of color Doppler interrogation of any intrauterine band-like structure.

## Case Report

A 33-year-old gravida 3 para 2 underwent a routine second-trimester anatomic survey at 19 ^6/7^ weeks’ gestation. Her pregnancy was otherwise uncomplicated, with low-risk noninvasive prenatal screening and no history of uterine instrumentation.

Ultrasound demonstrated a single live fetus with growth appropriate for gestational age. The placenta appeared right lateral, with placental tissue extending along both the anterior and posterior uterine surfaces. A thin, linear echogenic membrane was visualized within the lower uterine segment, spanning the anterior to posterior uterine wall without direct fetal involvement ( Fig. 1 ). No fetal structural abnormalities or evidence of limb entanglement were identified. The appearance initially raised concern for amniotic band syndrome.

**Fig. 1:**
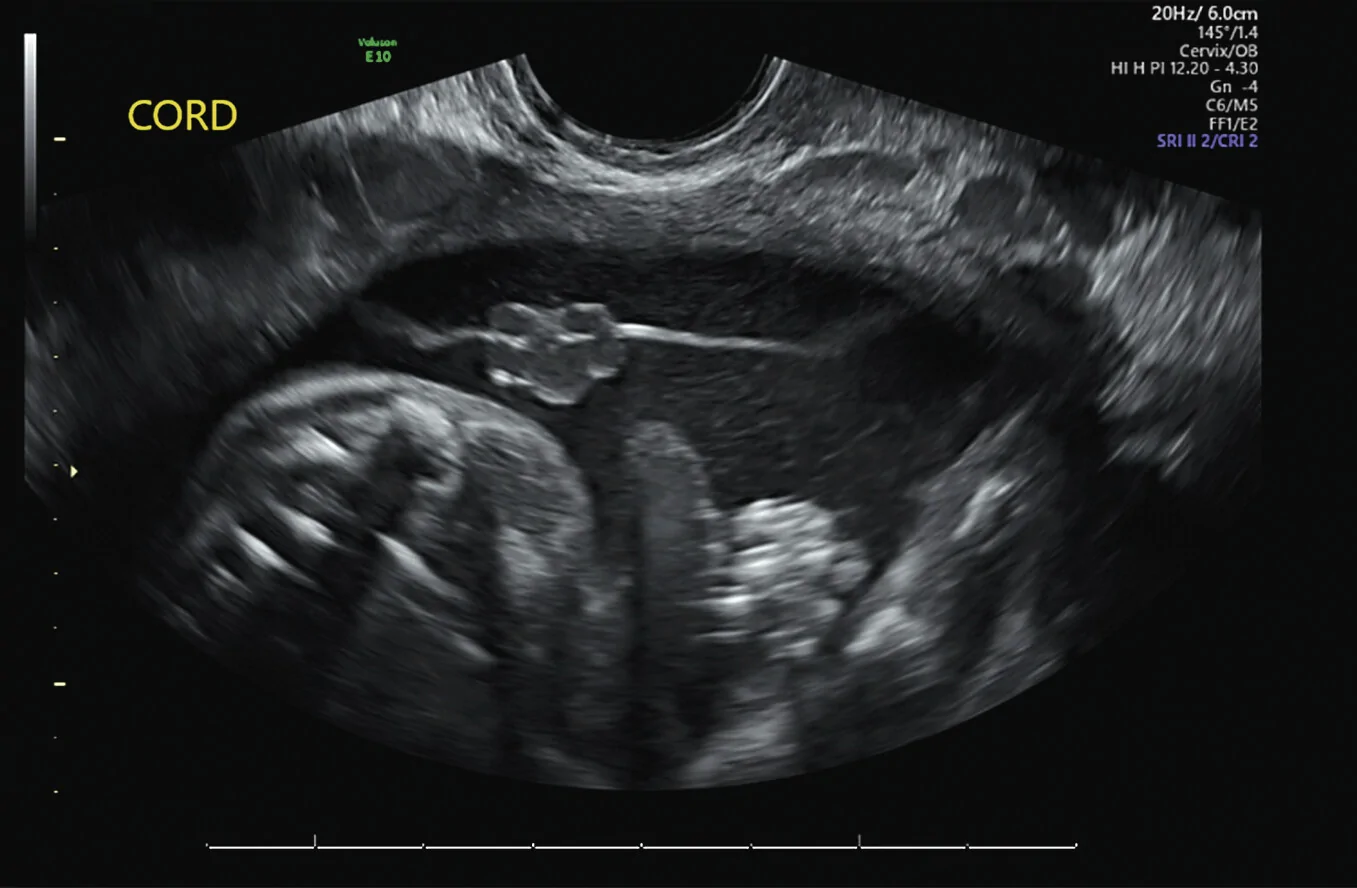
Transabdominal ultrasound at 19 ^6/7^ weeks demonstrating a thin intrauterine membrane (arrowhead) without fetal involvement.

Further evaluation demonstrated that the anterior and posterior placental components were connected by a membranous bridge. Color Doppler interrogation revealed fetal vessels coursing within this membrane ( Fig. 2 ), redirecting the differential diagnosis toward abnormal cord insertion rather than a true amniotic band. The umbilical cord appeared to traverse the membranous bridge before continuing toward the fetus, raising concern for exposed membranous fetal vessels and possible vasa previa. Targeted imaging demonstrated venous flow toward both placental components, with vessels coursing along the membranous structure and converging distally into a three-vessel cord ( Fig. 3 ).

**Fig. 2:**
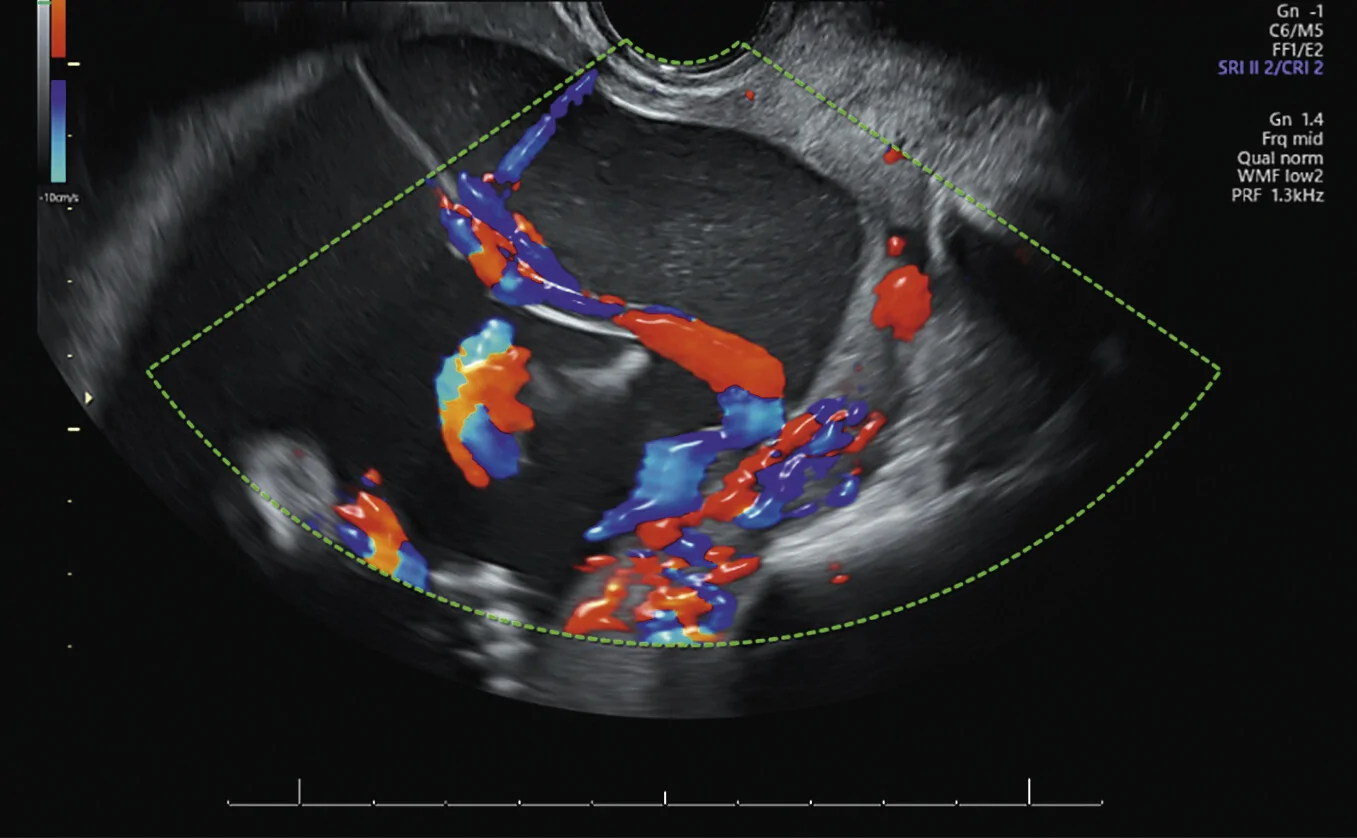
Transvaginal color Doppler ultrasound demonstrating fetal vessels coursing within a placental membrane (arrowhead), raising concern for abnormal cord insertion and vasa previa.

**Fig. 3:**
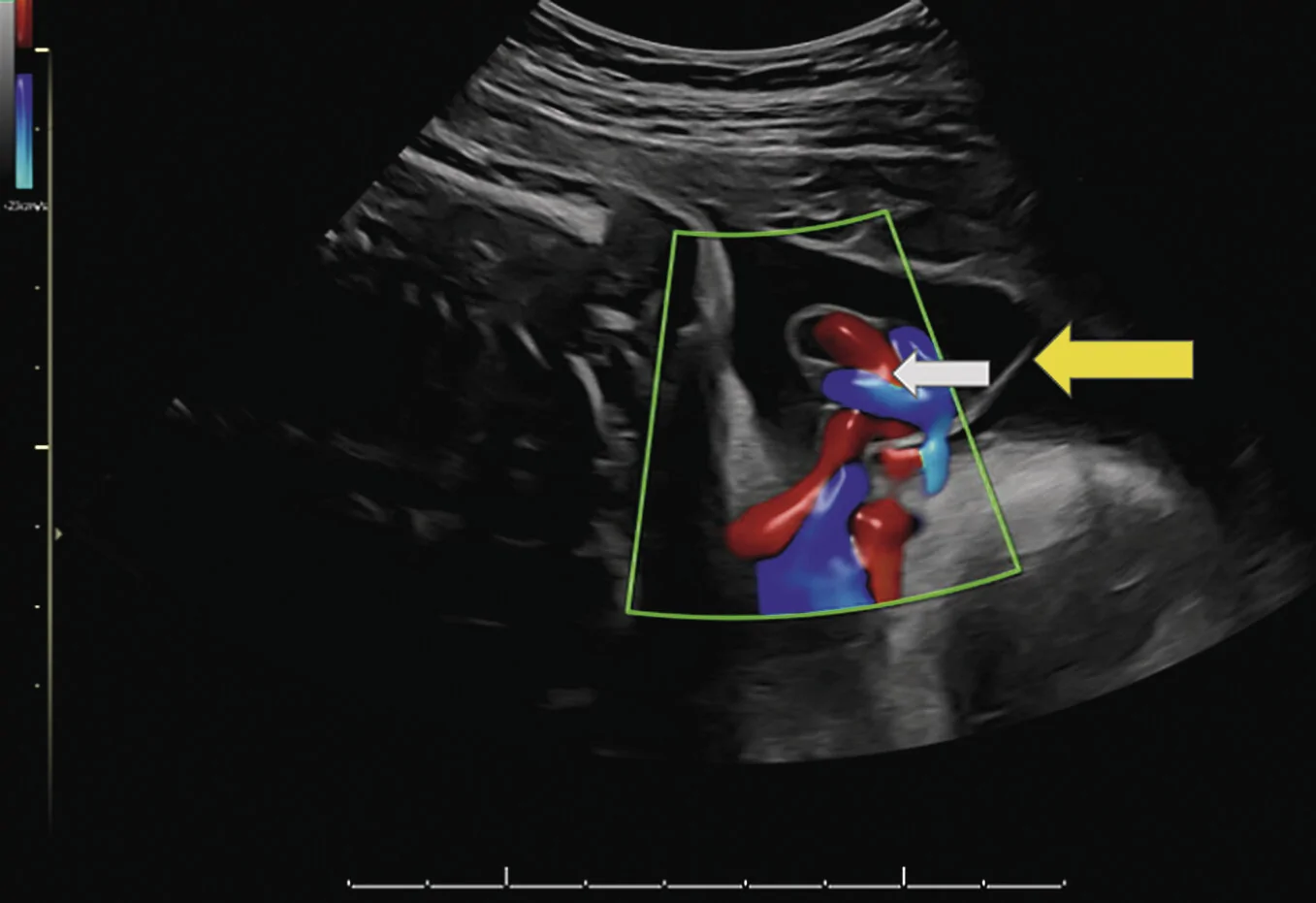
Cross-sectional transabdominal ultrasound at 27 weeks’ gestation demonstrating convergence of two umbilical veins (white arrow) originating from separate placental components and fusing into a three-vessel cord. A hyperechoic linear structure near the cervix (yellow arrow) represents the membranous bridge, which created the sonographic appearance of an amniotic band and raised concern for abnormal cord insertion.

Given the presence of membranous fetal vessels, transvaginal ultrasound with color Doppler was performed to assess the relationship of these vessels to the internal cervical os. Fetal vessels were visualized within the membrane but were located more than 3 cm from the internal cervical os, arguing against typical type I vasa previa. 4 Umbilical artery and venous Doppler waveforms remained normal throughout gestation.

Despite exclusion of vasa previa, concern remained for exposed fetal vessels lacking protection by Wharton’s jelly, raising the theoretical risk of vessel compression or rupture. 2 Given this diagnostic uncertainty and the absence of established management guidelines for suspected furcate cord insertion, serial fetal growth surveillance and antenatal testing, including nonstress tests and biophysical profiles, were initiated at 32 weeks’ gestation and remained reassuring.

A planned primary cesarean delivery was performed at 37 ^3/7^ weeks’ gestation to avoid potential labor-associated stress on exposed membranous vessels, including compression or rupture. Early-term delivery was selected to balance the theoretical risk of vessel injury against neonatal maturity. A healthy neonate was delivered with normal Apgar scores.

Gross placental examination confirmed a bilobed placenta connected by a thin placental bridge ( Fig. 4 ). Furcate separation of the umbilical vessels was identified, with the vessels branching prior to placental insertion and coursing along the amniotic membrane before inserting into the smaller placental lobe ( Fig. 5 ). Each placental lobe was supplied by a corresponding umbilical artery and vein inserting velamentously, and these vessels merged to form a three-vessel cord. These findings confirmed that the membranous structure visualized prenatally represented furcate cord insertion with exposed membranous fetal vessels. 3 Formal histopathologic examination was not performed, as the gross findings were considered clinically sufficient to explain the prenatal imaging and there were no additional maternal or neonatal indications for pathologic evaluation.

**Fig. 4:**
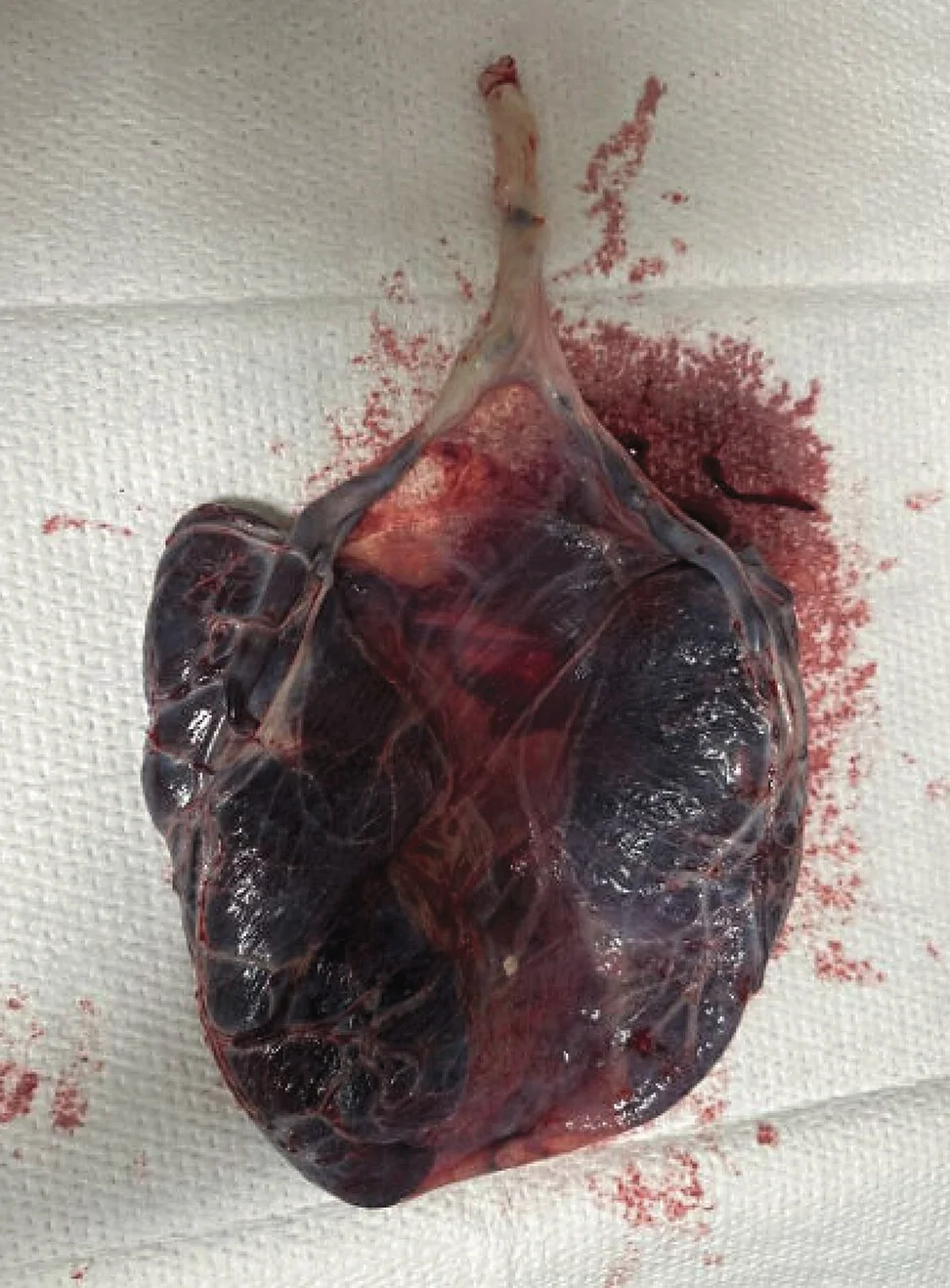
Gross examination of the bilobed placenta demonstrating two placental lobes connected by a thin placental bridge.

**Fig. 5:**
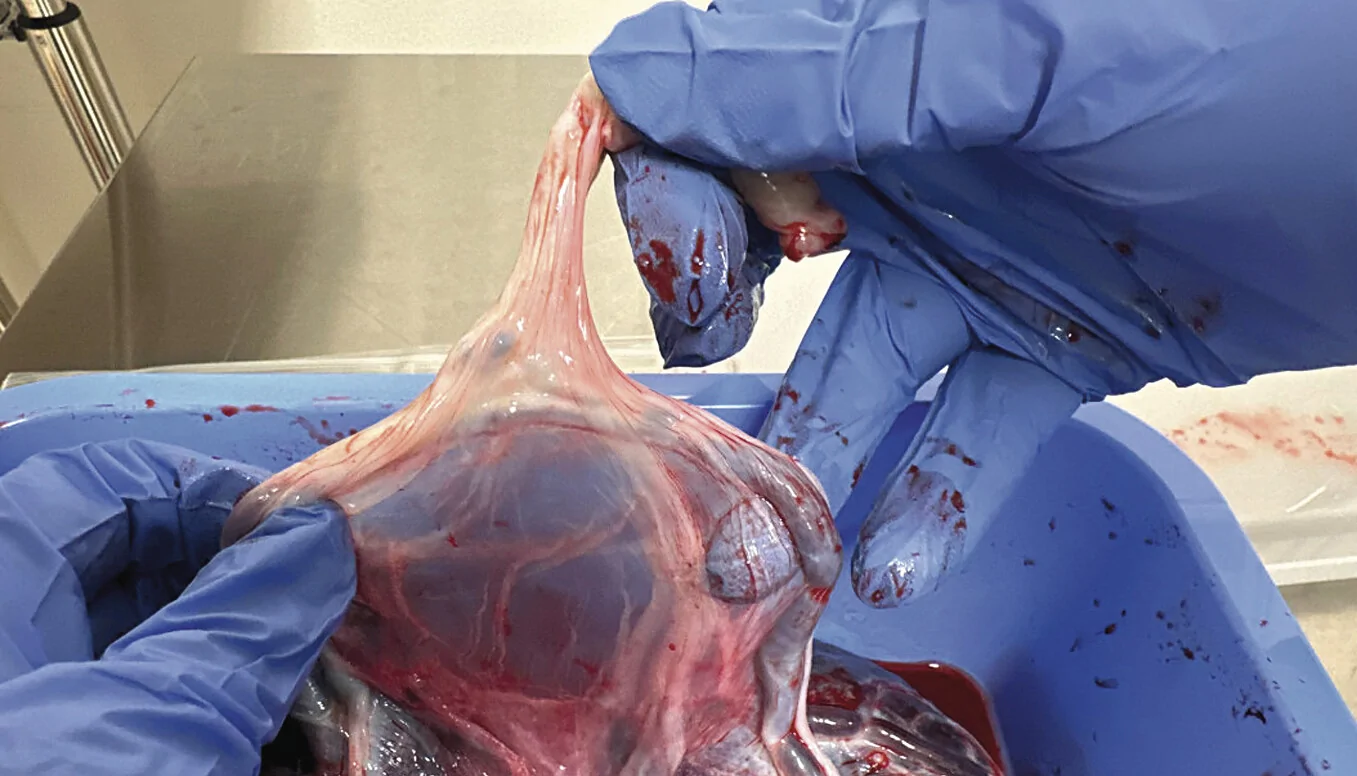
Gross examination demonstrating furcate separation of the umbilical vessels, with the vessels branching before placental insertion and coursing unsupported along the fetal membranes before velamentous insertion into the placental lobes.

## Discussion

Furcate umbilical cord insertion is a rare placental abnormality characterized by premature separation of the umbilical vessels before they reach the placental surface, with resultant loss of the protective Wharton’s jelly. Pathologic series estimate a prevalence of approximately 0.1 to 0.16% of pregnancies, but prenatal diagnosis is uncommon, and the condition is likely underrecognized. 1 2 The clinical significance lies in the exposure of unprotected fetal vessels, which may be vulnerable to compression or rupture; intrauterine fetal demise attributed to vessel rupture has been reported. 1

This case highlights an important diagnostic pitfall: membranous fetal vessels may mimic amniotic band syndrome on prenatal ultrasound. Linear echogenic intrauterine structures are often presumed to represent amniotic bands; however, true amniotic band involvement of the umbilical cord is rare, potentially catastrophic, and frequently diagnosed only after fetal demise. 5 6 In contrast, vascular structures associated with abnormal cord insertion may produce a similar band-like appearance on grayscale imaging. Vascularized intrauterine membranes may also mimic uterine synechiae with traversing vessels, further complicating differentiation from vasa previa. Failure to distinguish among these entities may result in either inappropriate reassurance or unnecessary concern. Color Doppler interrogation is, therefore, essential in the evaluation of any intrauterine band-like structure; in this case, Doppler imaging demonstrated fetal vessels within the membrane, effectively excluding a true amniotic band and redirecting the differential toward abnormal cord insertion. 7 8

Diagnostic complexity was further increased by subtle bilobed placental morphology, which was not clearly recognized prenatally. Bilobed placenta is associated with abnormal cord insertion in up to 27% of cases, and when the lobes are closely apposed, the connecting vascular bridge may obscure the underlying placental configuration. 9 In this case, the combination of bilobed placenta and furcate vessel separation created a membranous vascular structure that mimicked both amniotic band syndrome and vasa previa.

Although vasa previa was excluded by transvaginal Doppler assessment, concern remained for exposed membranous fetal vessels lacking Wharton’s jelly protection. In the absence of established guidelines for the management of suspected furcate cord insertion, a risk-based approach was adopted. Given the potential for vessel rupture or compression, analogous to the pathophysiology observed in velamentous cord insertion, serial surveillance and a planned early-term cesarean delivery were pursued to reduce the risk of catastrophic fetal hemorrhage. 10 11

This case underscores several clinical lessons. First, any intrauterine band-like structure should undergo careful Doppler interrogation to exclude a vascular etiology. Second, abnormal cord insertion should be considered when fetal vessels are identified within membranous structures, even in the absence of classic features of velamentous insertion or vasa previa. Finally, recognition of furcate cord insertion as a potential source of exposed fetal vessels may inform individualized management strategies in the absence of standardized guidelines.

## Data Availability

The underlying research data of this article are not publicly available due to patient privacy. The data are available from the corresponding author on reasonable request.

## Declaration of GenAI Use

In the preparation of this manuscript, the authors used a generative artificial intelligence (AI) large language model tool to assist with drafting, language editing, and formatting of the text (including the abstract, main text sections, and reference formatting). The AI tool was used solely as a writing and editing aid. The AI tool was not used to generate, analyze, or interpret clinical data; to create or alter any images or figures; to produce original scientific findings; or to identify or select references. All clinical content, data, interpretations, and conclusions originate from the authors. The authors reviewed, verified, and edited all AI-assisted text, independently confirmed the accuracy of all statements and references, and take full responsibility for the entire content of the manuscript, including any portions that were AI-assisted. No AI tool is listed as an author, in accordance with the authorship criteria of the International Committee of Medical Journal Editors (ICMJE), which require accountability that an AI cannot assume.
